# Cooperation between Engulfment Receptors: The Case of ABCA1 and MEGF10

**DOI:** 10.1371/journal.pone.0000120

**Published:** 2006-12-27

**Authors:** Yannick Hamon, Doriane Trompier, Zhong Ma, Victor Venegas, Matthieu Pophillat, Vincent Mignotte, Zheng Zhou, Giovanna Chimini

**Affiliations:** 1 Centre d'Immunologie de Marseille Luminy, Institut National de la Santé et de la Recherche Médicale, Centre National de la Recherche Scientifique, Université de la Méditerranée, Marseille, France; 2 Carter Immunology Center and the Department Of Microbiology, University of Virginia, Charlottesville, Virginia, United States of America; 3 Verna and Marrs McLean Department of Biochemistry and Molecular Biology, Baylor College of Medicine, Houston, Texas, United States of America; 4 Institut Cochin, Institut National de la Santé et de la Recherche Médicale U 567, Centre National de la Recherche Scientifique UMR 8104, Université Paris V, Paris, France; The University of Birmingham, United Kingdom

## Abstract

The engulfment of dying cells is a specialized form of phagocytosis that is extremely conserved across evolution. In the worm, it is genetically controlled by two parallel pathways, which are only partially reconstituted in mammals. We focused on the recapitulation of the CED-1 defined pathway in mammalian systems. We first explored and validated MEGF10, a novel receptor bearing striking structural similarities to CED-1, as a *bona fide* functional ortholog in mammals and hence progressed toward the analysis of molecular interactions along the corresponding pathway. We ascertained that, in a system of forced expression by transfection, MEGF10 function can be modulated by the ATP binding cassette transporter ABCA1, ortholog to CED-7. Indeed, the coexpression of either a functional or a mutant ABCA1 exerted a transdominant positive or negative modulation on the MEGF10-dependent engulfment. The combined use of biochemical and biophysical approaches indicated that this functional cooperation relies on the alternate association of these receptors with a common partner, endogenously expressed in our cell system. We provide the first working model structuring in mammals the CED-1 dependent pathway.

## Introduction

The engulfment of dying cells is ruled by the concerted action of several molecules [1]: they act either at the cell surface to recognize the prey that is to be engulfed, or intracellularly to activate signalling cascades leading to the required spreading of the membrane during ingestion. Extensive genetic approaches in *C. elegans* have highlighted that engulfment genes, collectively belonging to the *ced* group (cell death abnormal) [2], act along two distinct and parallel pathways converging towards the same end-effectors. CED-2, CED-5, CED-10 and CED-12 act in the first pathway, whereas CED-1, CED-6 and CED-7 identify the second [1]. CED-10 is Rac-1, a small GTPase able to induce actin polymerization, which is an essential final step in phagocytosis, and acts in both signalling pathways [3]. Recently, the large GTPase dynamin has been shown to mediate the signalling of the phagocytic receptor CED-1 and promote membrane renewal at the site of ingestion of corpses [4].

Mammalian orthologs to the ced genes have been identified along time mostly on the basis of sequence homology, and then further validated as engulfment controlling genes in appropriate cellular systems. Namely the CED-2 pathway corresponds, in mammals, to the membrane recruitment of Dock180, CrkII and ELMO triggered by the occupancy of integrin αv β5 [5], [6]. Interestingly, the membrane receptor orchestrating this signalling cascade in the nematode remains still elusive. Small GTP binding proteins of the Rac subfamily act downstream in the cascade and lead to actin polymerization and pseudopod extension in both nematodes and mammals [7]. The interactions between the proteins belonging to the CED-1 pathway are less well established both in the mammalian and nematode systems [8]. In fact, though CED-6 [9] and its mammalian ortholog GULP are known to dimerize and are able to interact with CED-1 through phosphorylatable tyrosine residues in the NPxY motif [10], [11], no clear definition of the role of the ATP binding cassette transporters (CED-7/ABCA1) has so far been achieved [12]–[14]. ABCA1 functions as a lipid translocator [15], [16] and favours engulfment by inducing local modifications of the membrane composition in phospholipids. Indeed, the membrane lipid composition could instruct both the lateral mobility or clustering of receptors at contact sites and the recruitment of dynamin to forming phagosomes [17]. Consistently, formal evidence of the requirement of CED-7 for the recruitment of CED-1 around engulfed corpses has been provided [18]. However, the modalities of molecular interactions, if any, between CED-1 and CED-7 have not been addressed.

CED-1 is so far the only membrane receptor identified as an engulfment gene in the nematode. This contrasts with the mammalian system where a plethora of surface molecules have been implicated in the process [19]. Some of them have been proposed as CED-1 orthologs but none has been explicitly assigned as yet. On the basis of interaction analysis, CD91/LRP-1 is a consistent candidate, in spite of its broad substrate recognition [11] and its weak architectural conservation. Recently, MEGF10 has emerged as a protein structurally related to CED-1 [20]. No functional role has been assigned to MEGF10 so far. In this paper, we explore and validate its function as an engulfment receptor by providing experimental evidence in both *C. elegans* and mammalian systems. In addition, by the combined use of cellular and biochemical approaches we provide evidence that ABCA1 and MEGF10 interact at the molecular level. This allows us to propose, for the first time, a framework model of interactions structuring the whole CED-1 dependent engulfment pathway.

## Results

### MEGF10 is a candidate CED-1 ortholog in mammals

**Figure 1:**
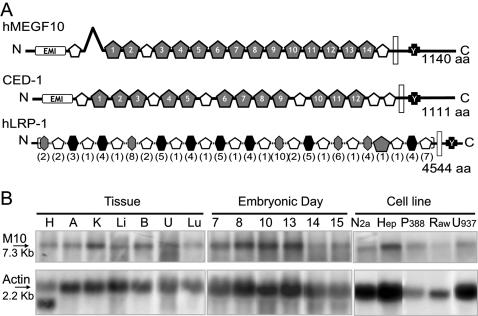
MEGF10 is a candidate CED-1 ortholog in mammals: structural features and expression pattern. **(A) Schematic drawing comparing the molecular architecture of human MEGF10 and CED-1.** MEGF10 and CED-1 share a highly similar structural pattern. Structural predictions are based on sequence analysis by the Geneworks software and on the SMART website (http://smart.embl-heidelberg.de/smart/show_motifs.pl). The cysteine-rich domains in both proteins were analyzed in term of sequential conservation of EGF-like domains, either 6-Cys EGF-like domains (white pentagons) or laminin-type EGF-like domains (gray pentagons). The Emilin domains [20] (EMI rectangles), the predicted transmembrane segments (white rectangles) and the NPxY motif (black crosses) implicated in the molecular interactions with GULP/CED-6, are also indicated. For comparison a schematic illustration of the predicted structure of LRP-1 is shown; low density lipoprotein receptor domains are indicated by hexagons, in brackets is indicated the number of repetitions. **(B) MEGF10 expression pattern.** Northern blot analysis of MEGF10 transcript (M10: 7.3 Kb) in RNA samples prepared from adult mouse tissues (H: Heart, A: Adrenals, K: Kidney, Li: Liver, B: Brain, U: Uterus, Lu: Lung), whole mouse embryos (Embryonic day 7, 8, 10, 13, 14 and 15) and a panel of cell lines (Neuro 2a – mouse neuroblastoma, HepG2 – human hepatoma, P388D1, Raw 264.7 and U937 – mouse and human macrophages respectively). Membranes were simultaneously hybridized with an actin probe as a quantitation control.

The analysis of human MEGF10 sequence and of its structure predictions highlighted unquestionable and stringent similarities with CED-1 (Figure 1A). Indeed, both molecules display in succession, from N- to C-terminus, an Emilin (EMI) domain, 17 and 18 (for MEGF10 and CED-1, respectively) canonical or atypical 6-cysteine EGF domains, a transmembrane domain, and the NPxY module for potential tyrosine phosphorylation. CED-1 possesses an additional potential site for tyrosine phosphorylation, an YxxL module, which is absent in MEGF10. In functional terms the EGF-like domains are presumed to be implicated in the binding to, yet unknown, extracellular ligands, and the phosphorylatable tyrosine in the NPxY site has been proven crucial for both the CED-1 mediated signal transduction and its interaction with the phosphotyrosine binding (PTB) domain of the adaptor CED-6 [11], [18].

On the basis of these striking similarities, we checked the ability of MEGF10 to promote engulfment in mammalian systems. As a first step, we analyzed its expression pattern by Northern blot on a panel of RNA samples originating from tissues and cell lines. As shown in Figure 1B, a specific MEGF10 transcript, of approximately 7.3 kb in size, was widely expressed, being undetectable only in CHO, COS-7 and HeLa cells (not shown). Its presence during development, in adult tissues and in macrophage cell lines is consistent with the proposed function during the engulfment of dead cells.

**Figure 2:**
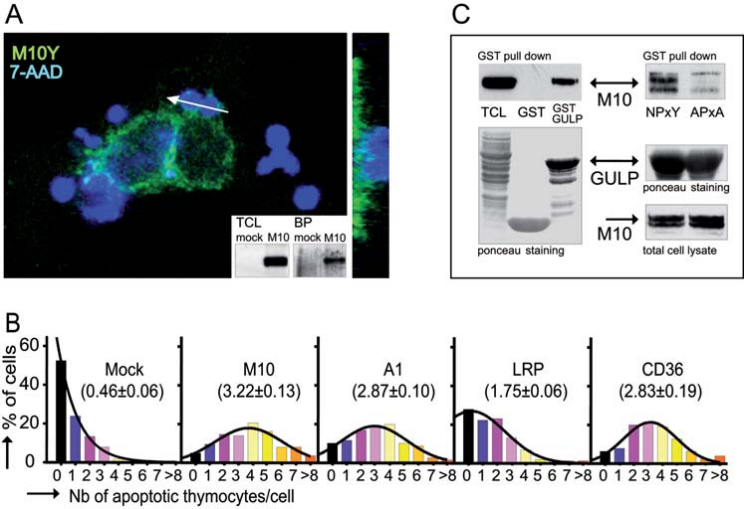
Functional assessment of MEGF10 as an engulfment receptor. **(A) MEGF10 is expressed at the cell surface and clusters around cell corpses during engulfment.** Confocal optical X-Y sections show the localization of MEGF10 EYFP (M10Y - pseudo color green) in transfected HeLa cells challenged with 7-AAD labelled apoptotic thymocytes (pseudo color blue). The white arrow indicates the location of the X-Z plan shown on the right. The surface localization was confirmed by the analysis of surface biotinylation as shown in the insert. Western blot of total cell lysates probed with an anti-GFP antibody (TCL, left panel) are compared to blots of biotinylated proteins (BP, right panel). Mock transfected cells (mock), and MEGF10 EYFP transfected (M10) HeLa cells. **(B) The expression of MEGF10 enhances the phagocytic ability of HeLa Cells.** *In vitro* phagocytosis assays were carried out on HeLa cells transfected with the indicated engulfment receptors. Results are expressed as distribution histograms. Percentages of cells, scored on ≥100 transfected cells, are plotted against the number of tethered/ engulfed apoptotic thymocytes. The phagocytic index, computed out of at least 4 individual experiments, is indicated in brackets. Mock: mock transfected cells; M10: MEGF10; A1: ABCA1; LRP: LRP-1. **(C) MEGF10 can interact with GULP as assessed by GST pull down.** Left panel: lysates of HeLa cells transfected with MEGF10 EYFP were incubated with bacterially produced GST or GST-GULP fusion protein before fractionation on SDS-PAGE and blotting onto nitrocellulose membrane. Total proteins were visualized by Ponceau S staining (lower panel) whereas MEGF10 binding to GULP was detected by hybridization with an anti-GFP antibody (upper panel –TCL: Total cell lysate). Right panel: GULP and MEGF10 interact via the NPxY motif since its mutation to APxA abrogates binding (upper panel - NPxY: MEGF10 EYFP-NPxY, APxA: MEGF10 EYFP-APxA). Equivalent amounts of bacterially produced GST-GULP (middle panel) were incubated with equivalent amounts of MEGF10 expressed in transfected cells (lower panel). MEGF10 detection was performed by probing with an anti-GFP antibody on total cell lysate (lower panel) or on pulled down samples (upper panel).

MEGF10 was expressed by transient transfection in HeLa cells as a chimera with EGFP or its variants (ECFP and EYFP), grafted as a C-terminal tailpiece. Upon transfection, MEGF10 was translated as 170 kDa protein targeted to the plasma membrane as shown by the surface biotinylation of the immunoprecipitated protein (Figure 2A, insert) and confirmed by confocal analysis (Figure 2A). In addition, upon challenge with apoptotic thymocytes, an enrichment of MEGF10 at the sites of contact with prey was detectable (Figure 2A, arrow).

The functional involvement of MEGF10 in the engulfment process was further confirmed by the modification of phagocytic ability induced by its expression in HeLa cells. Indeed, a significant engulfment competence followed MEGF10 expression (Figure 2B), as indicated by a phagocytic index (average number of prey ingested per transfected cell) comparable to that elicited by ABCA1 or CD36 [21] and in fact higher than that induced by LRP-1, a previously suggested CED-1 ortholog in mammals [11].

To strengthen the identification of MEGF10 as a CED-1 ortholog, we examined the molecular interactions with GULP [22], [23], the mammalian ortholog of CED-6. We thus investigated by pull down assays whether molecular contacts existed between MEGF10 and GULP as well as their dependence on the NPxY motifs, previously shown to be involved in CED-1/CED-6 and GULP/LRP-1 contacts [11], [24]. As shown in Figure 2C, bacterially produced GST-GULP specifically interacted with MEGF10 expressed in HeLa cells upon transfection. Similarly, endogenously produced GULP coprecipitated with MEGF10 in COS-7 cells transfected with both constructs (not shown). The loss of interaction upon mutation of the NPxY motif in MEGF10 indicated that these residues mediate the molecular contact between the receptor and the GULP adaptor (Figure 2C), as should be expected in the case of orthology. These data thus provide compelling functional evidence that MEGF10 can act as an engulfment receptor in reconstituted systems and designate this protein as a *bona fide* candidate CED-1 ortholog.

To further seek evidence that MEGF10 acts as a phagocytic receptor we examined whether MEGF10 could rescue the defects of *ced-1* mutants in the engulfment of apoptotic cells in *C. elegans*. We generated transgenic worms with a construct driving the expression of MEGF10 in engulfing cells under the control of CED-1 promoter [18]. The MEGF10::GFP fusion protein under the control of the *ced-1* promoter was efficiently translated and observed at the cell surface; however, in most lines, the localization of the fusion protein displayed an additional punctuated pattern (not shown). This was suggestive of the formation of intracellular aggregates and indicated that the heterologous expression ensued in inefficient trafficking to the plasma membrane.

We scored the number of cell corpses in the head of newly hatched L1 larvae as a measurement of the defect in engulfment. In wild type animals, no cell corpses remain at this stage due to swift engulfment and degradation occurring during embryogenesis. In *ced-1(e1735)* mutant animals, close to 30 persistent cell corpses were observed (Table 1). As previously observed, the expression of P*_ced-1_ ced-1::gfp*, the positive control construct, completely rescued the engulfment defect of the *ced-1(e1735)* mutants (Table 1) [18], indicating that a GFP fusion to the C-terminus of CED-1 does not affect its function. Expression of human MEGF10::GFP fusion in engulfing cells (P*_ced-1_ megf10::gfp*) partially rescued the engulfment defect of *ced-1(e1735)* (Table 1). The number of persistent cell corpses in these transgenic animals is about 69–74% of that of the non-transgenic animals (three independent lines assayed). In addition, the expression of P*_ced-1_ megf10::gfp* in wild type animals did not result in the accumulation of cell corpses (Table 1), indicating that MEGF10 did not exert any dominant-negative effect on engulfment. These results indicate that MEGF10 can partially replace CED-1 as an engulfment receptor in the nematode. Collectively the above results provide strong evidence that MEGF10 can be considered a *bona fide* *ced-1* ortholog in mammals.

**Table 1:**
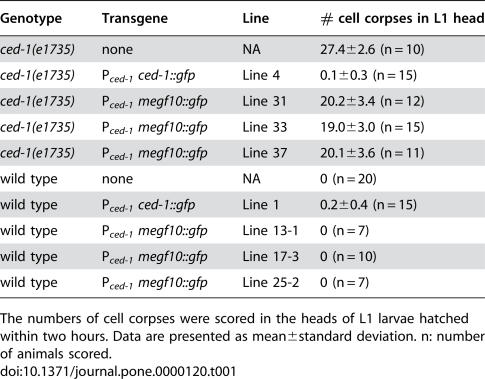
Human MEGF10 can partially replace *ced-1* function in the engulfment of apoptotic cells in *C. elegans*.

Genotype	Transgene	Line	# cell corpses in L1 head
*ced-1(e1735)*	none	NA	27.4±2.6 (n = 10)
*ced-1(e1735)*	P*_ced-1_* *ced-1::gfp*	Line 4	0.1±0.3 (n = 15)
*ced-1(e1735)*	P*_ced-1_* *megf10::gfp*	Line 31	20.2±3.4 (n = 12)
*ced-1(e1735)*	P*_ced-1_* *megf10::gfp*	Line 33	19.0±3.0 (n = 15)
*ced-1(e1735)*	P*_ced-1_* *megf10::gfp*	Line 37	20.1±3.6 (n = 11)
wild type	none	NA	0 (n = 20)
wild type	P*_ced-1_* *ced-1::gfp*	Line 1	0.2±0.4 (n = 15)
wild type	P*_ced-1_* *megf10::gfp*	Line 13-1	0 (n = 7)
wild type	P*_ced-1_* *megf10::gfp*	Line 17-3	0 (n = 10)
wild type	P*_ced-1_* *megf10::gfp*	Line 25-2	0 (n = 7)

The numbers of cell corpses were scored in the heads of L1 larvae hatched within two hours. Data are presented as mean±standard deviation. n: number of animals scored.

### ABCA1 and MEGF10 interact during engulfment

**Figure 3:**
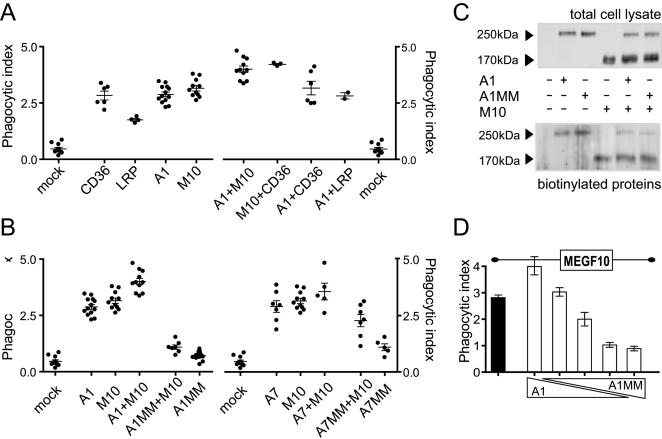
ABCA1 and MEGF10 cooperation during engulfment. **(A) Positive cooperation between engulfment receptors.** Phagocytosis assays were performed on HeLa cells transfected with the indicated molecules. Phagocytic Indexes (PI)±SEM, and number of independent experiments (n) are as follows : Mock: Mock transfected cells PI = 0.46±0.06, n = 11, CD36 PI = 2.83±0.19, n = 6, LRP: LRP1 PI = 1.75±0.06, n = 4, A1: ABCA1 PI = 2.87±0.11, n = 13, M10: MEGF10 PI = 3.15±0.12, n = 11, A1+M10: ABCA1+MEGF10 PI = 4.00±0.14, n = 11, M10+CD36: MEGF10+CD36 PI = 4.22±0.04, n = 3, A1+CD36: ABCA1+CD36 PI = 3.16±0.30, n = 6, A1+LRP: ABCA1+LRP1 PI = 2.82±0.14, n = 2. Coexpressions of receptors were performed by transfection of equal quantities of plasmid cDNA of each species. ECFP or EYFP fusion proteins were used and visualized by confocal microscopy. **(B) The inactive ABCA1MM mutant hampers the ability of MEGF10 to promote engulfment.** Coexpression of MEGF10 and ABCA1MM (A1MM+M10) reverts the phagocytic index to background levels. The reversion is specific since the coexpression of MEGF10 with ABCA7 (A7) or its inactive mutant (A7MM) is devoid of significant effect on the phagocytic index. Phagocytosis assays were performed on HeLa cells transfected with the indicated molecules. A1MM+M10: ABCA1MM+MEGF10 PI = 1.09±0.10, n = 7, A1MM: ABCA1MM PI = 0.71±0.05, n = 13, A7: ABCA7 PI = 2.90±0.26, n = 7, A7+M10: ABCA7+MEGF10 PI = 3.57±0.37, n = 5, A7MM+M10: ABCA7MM+MEGF10 PI = 2.28±0.27, n = 7, A7MM: ABCA7MM PI = 1.1±0.15, n = 5. ECFP or EYFP fusion proteins were used and visualized by confocal microscopy. **(C) ABCA1MM does not affect the trafficking of MEGF10 to the cell surface.** Western blot of lysates from cells expressing MEGF10 and ABCA1 or its inactive mutant ABCA1MM were probed with an anti-GFP antibody (upper panel) and compared to blots of biotinylated proteins (lower panel). Mock transfected cells are compared to cells transfected with ABCA1 EYFP (A1), ABCA1MM EYFP (A1MM), MEGF10 ECFP (M10), or the indicated combinations. **(D) Molecular competition between wild type and mutant transporter underlies the ABCA1MM effect.** ABCA1MM expression levels titrate the ABCA1/MEGF10 cooperation during engulfment. Various ratios of ABCA1/ABCA1MM cDNA (5/0; 4/1; 2.5/2.5; 1/4 and 0/5 from the left to the right) were cotransfected with fixed amounts of MEGF10 and phagocytic assays performed on transfected cells. The phagocytic index is expressed as average of 3 independent experiments±SEM. Values are from the left to the right: MEGF10: 2.81±0.10; 3.99±0.31; 3.03±0.16; 2.00±0.26; 1.02±0.09; 0.89±0.09.

To gain insight in the cooperativity between engulfment receptors, and with the aim of assigning mammalian receptors to individual pathways recapitulating those in the nematode, we set out to analyze the phenotype conferred by the simultaneous expression of various combinations of engulfment receptors (Figure 3A). Coexpression of both MEGF10 and ABCA1 significantly increased the phagocytic index (4.00±0.14, n = 11) with respect to single transfected cells (P<0.0001 vs. ABCA1 2.87±0.11, n = 13, P<0.01 vs. MEGF10 3.15±0.12, n = 11). MEGF10 and CD36 together produced a similar effect (4.22±0.04 n = 3, P<0.001 vs. MEGF10), whereas ABCA1 and CD36 coexpression led to a minimal enhancement when compared to single transfected cells (3.16±0.3, n = 6 versus the single CD36 2.83±0.19, n = 6). This clearly illustrated the presence of an additive effect of MEGF10 over both CD36 and ABCA1, but did not allow the clear sorting of individual receptors to distinct engulfment pathway. However, the limited effect observed after the coexpression of ABCA1 and CD36 prompted us to consider that CD36 could act independently from the ABCA1 facilitator function whereas MEGF10 appeared highly sensitive to the presence of the transporter. To validate this hypothesis we took advantage of an ATPase less ABCA1 mutant (MM) [14], [25]. The substitutions of crucial Lys into Met in the nucleotide binding domains (NBD) carried by MM (position 939 and 1952) are known to lead to a complete loss of function of the ABCA1 transporter without affecting its trafficking. In the context of engulfment, the expression of ABCA1MM prevented any increase in the phagocytic ability of HeLa cells (0.71±0.05, n = 13 versus mock transfected cells 0.46±0.06, n = 11; Figure 3). Moreover, the coexpression of ABCA1MM with MEGF10 in HeLa recipients completely reverted the MEGF10-induced engulfment to background levels (Figure 3B; 1.09±0.1, n = 7, P<0.0001 vs. MEGF10) thus showing that a transdominant negative effect is exerted on MEGF10 by the mutant ABCA1.

The effect was specific to ABCA1; indeed in a similar experimental set up, ABCA7 [26], [27], the closest ABCA1 relative in the A class of ABC transporters, exerted little influence on the phagocytic behaviour of recipient cells. In HeLa cells, we transiently coexpressed MEGF10 and ABCA7 or its mutant form ABCA7MM that mirrors the ABCA1MM forms and bears Lys to Met substitutions in both NBDs. In our cell systems, where both forms of the ABCA7 transporter decorate the cell membrane, we failed to observe either additive or transdominant negative effects over the MEGF10-induced engulfment competence (Figure 3B).

These results suggested that specific molecular interactions between ABCA1 and MEGF10 could underlie the transdominant effect of ABCA1MM. We first ruled out any effect of ABCA1MM on the intracellular trafficking of MEGF10 by the analysis of surface biotinylation of MEGF10 coexpressed with either ABCA1 or its mutant (Figure 3C). We further validated that the transdominant negative effect reflected molecular competition. Indeed, the inhibition could be efficiently titrated by varying the molar ratios (Figure 3D) of wild type versus mutant forms of the ABCA1 transporter coexpressed in HeLa cells with fixed amounts of MEGF10.

**Figure 4:**
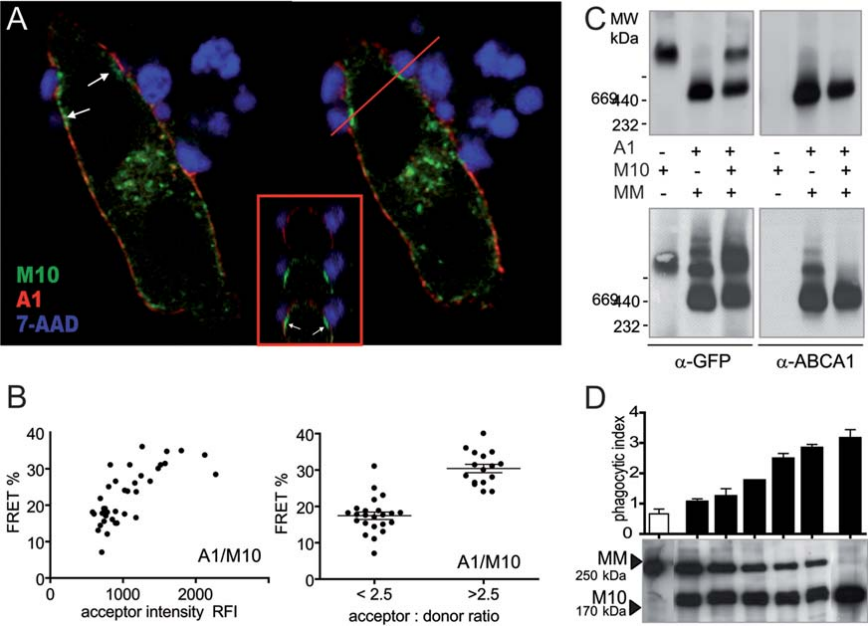
Assessment of molecular interactions between MEGF10 and ABCA1. **(A) MEGF10 and ABCA1 distribute differentially during engulfment.** X-Y confocal sections of transfected HeLa cells evidence that during the engulfment of apoptotic thymocytes (7-AAD in pseudo color blue) MEGF10 (M10: MEGF10 ECFP in green) is recruited at the bottom of the forming phagocytic cup (arrows) while ABCA1 stays at its edges (A1: ABCA1 EYFP in red). X-Z sections of the regions highlighted by the red line confirm the spatial distribution of MEGF10 in the core of the phagocytic cup. **(B) Molecular proximity between ABCA1 and MEGF10 assessed by FRET.** Imaging FRET analysis of cells expressing simultaneously ABCA1 EYFP and MEGF10 ECFP indicates that the C terminal tailpieces lie at a distance of <100 Å. Transfer efficiency is insensitive to acceptor intensity (YFP RFI – left panel) but sensitive to acceptor: donor ratios (right panel). **(C) MEGF10 hampers the oligomerization of ABCA1.** Native PAGE profile of lysates of HeLa cells transfected with equivalent amounts of MEGF10 and either ABCA1 (A1 upper panel) or its mutant ABCA1MM (MM lower panel). The immunodetection with anti-GFP and anti-ABCA1 antibodies reveals no evidence of comigration of the two proteins in a single complex in the case of transfection with the wild type functional transporter. However the presence of MEGF10 greatly destabilises the pattern of oligomerization linked to the expression of ABCA1MM. **(D) Titration of the transdominant negative effect of ABCA1MM on MEGF10.** Cells were transfected with various ratios of ABCA1MM and MEGF10 as shown by the SDS-PAGE pattern (lower panel) and analyzed by phagocytic assays and native PAGE (not shown). Increased ratios of MEGF10 over ABCA1MM lead to increased destabilization of ABCA1MM complexes (not shown) and rescue of phagocytic competence. Phagocytic indexes (averaged from 3 independent experiments)±SEM are from the left to the right: 0.66±0.16, 1.04±0.11, 1.23±0.27, 1.75±0.02, 2.47±0.19, 2.82±0.13, 3.14±0.30.

We then used imaging FRET as an appropriate technique to assess close molecular proximity between ABCA1 and MEGF10 [25]. We measured the efficiency of energy transfer occurring between ABCA1 EYFP and MEGF10 ECFP simultaneously expressed in HeLa cells by the method of acceptor photobleaching [28], [29]. We analyzed the transfer of energy occurring in the absence of any phagocytic challenges since confocal analysis had shown that, while colocalized at rest, ABCA1 and MEGF10 distributed differentially during phagocytosis (Figure 4A). In fact ABCA1 was consistently enriched at the edges of the phagocytic cup, whereas MEGF10 was dynamically recruited at its rim. Under these conditions, imaging FRET measurements indicated significant transfer of energy between the two molecules at the cell membrane (Figure 4B). The transfer was further validated as being due to molecular proximity rather than random collision by assessing its insensitivity to acceptor density, estimated as RFI of EYFP, but sensitivity to variation in the acceptor: donor ratio. Our measurements thus indicated that, when MEGF10 and ABCA1 are coexpressed at the plasma membrane, their fluorescent tailpieces lie at distance <100 Å.

To strengthen the evidence of interactions between the two molecules, we took advantage of the experimental conditions and tools developed to assess the oligomerization states occurring as part of the ATPase cycle of the ABCA1 transporter. Whole lysates of HeLa cells, expressing MEGF10 and either a functional ABCA1 or its ABCA1MM mutant, were fractionated by native PAGE and analyzed by immunoblotting with specific antibodies [25], [30]. No evidence of comigration of ABCA1 and MEGF10 proteins was found thus excluding a stable assembly in a single complex (Figure 4C, upper panel). However, we observed that the presence of MEGF10 destabilized the ATP induced oligomerization of ABCA1, which can be visualized experimentally by the use of the ABCA1MM mutant (Figure 4C, lower panel). This suggested that ABCA1 and MEGF10 share a common partner transiently shuttling between the transporter and the receptor. To validate this hypothesis, we analyzed the effect of titrating the amounts of MEGF10 versus ABCA1MM expressed in the cells; in fact if the two molecules compete for the same rate limiting partner, we should expect, and, indeed found, a direct correlation between destabilization of ABCA1 oligomers (not shown) and rescue of phagocytic competence (Figure 4D) at increasing ratios of MEGF10 over ABCA1MM. Indeed, in the presence of excess MEGF10, the phagocytic index of transfected HeLa cells reverted to the values observed in the presence of MEGF10 alone (Figure 4D).

## Discussion

In this report we provide evidence for MEGF10 as a *bona fide* ortholog of the nematode engulfment receptor CED-1. The evidence, which was initially born from structural considerations [20], was supported by functional studies in both the mammalian and nematode systems. Indeed, MEGF10 induced engulfment competence in the non phagocytic HeLa cells to indexes similar to those of the established engulfment receptors, CD36, ABCA1 and LRP-1. MEGF10 was also able to rescue the engulfment defect of *ced-1* mutant worms. It is worth noting however that the rescue of the *ced-1* phenotype appeared incomplete. This may be due to the observed defective intracellular trafficking of the MEGF10 receptor in the worm; in fact failure to efficiently reach the plasma membrane decreases *in situ* availability of MEGF10 with consequent reduced rescue of the engulfment defect. Of note the reciprocal experiment of heterologous expression of CED-1 in mammalian cells leads invariably to a massive retention in the endoplasmic reticulum (our observations and [11]). Thus, both CED-1 and its mammalian ortholog, in our hands MEGF10, might require species-specific chaperones to assist their folding and cellular trafficking.

Having validated MEGF10 as a CED-1 ortholog, we attempted to recapitulate in mammalian cells the molecular cascade of the CED-1-engulfment pathway. While we were unable to unambiguously assign receptors to a given engulfment pathway, we observed that the function of MEGF10 as an engulfment receptor was highly sensitive to the presence of ABCA1. Indeed, its coexpression with a functional ABCA1 led to enhanced engulfment competence, accompanied morphologically by an ABCA1-dependent redistribution of MEGF10 along the rims of the phagocytic cup. These data, which recapitulate the seminal observation in *C. elegans* on the CED-7/CED-1 relationship [18], are highly suggestive of transient molecular interactions between the two molecules. Likewise, we observed that the coexpression of MEGF10 with the mutant ABCA1MM induced a transdominant negative effect on the MEGF10-dependent engulfment competence.

To explain this phenomenon we explored several possibilities. We could rule out non specific effects due to impairment of intracellular trafficking of MEGF10 to the plasma membrane in the presence of the mutant ABCA1 or to the mere presence at the plasma membrane of ATP Binding Cassette molecules acting as modifiers of lipid packing/distribution. We in fact analyzed the behaviour of ABCA7, a close relative of ABCA1 that is able to drive cellular lipid effluxes [31]–[33], and whose involvement in engulfment has been recently suggested [34]. In our hands, the expression of ABCA7 at the surface of HeLa cells was devoid of cooperative effects on engulfment: while a general effect on the phagocytic competence could be observed, no transdominant effects (either positive or negative) over the MEGF10 induced phenotypes were detected. Conversely, we provided evidence for interactions between the ABCA1 transporter and MEGF10 by two methods relying on biophysical and biochemical approaches. We detected intermolecular transfer of energy via imaging FRET and observed by electrophoresis in native conditions that MEGF10 destabilized the oligomeric assemblies of the ABCA1 transporter. Increased ratios of MEGF10 over ABCA1MM were directly correlated to destabilization and to loss of the transdominant negative effect thus indicating competition for a single molecule. The limiting availability of this shared partner in HeLa cells provides the molecular rationale for the transdominant effect on both oligomerization and engulfment.

On the basis of these observations and of the previously gathered information on ABCA1 architecture and function [25] we propose the following model of the molecular interactions taking place. At the site of engulfment, ABCA1 molecules are activated, by yet unknown mechanisms linked to the recognition of the prey, and then enter the catalytic cycle. The binding of ATP induces assembly into complex oligomers containing ABCA1 multimers and additional molecules endogenously expressed in HeLa cells. ATP hydrolysis at the NBDs triggers local remodelling of lipid composition, such as the ABCA1 dependent outward displacement of phosphatidylserine [14], [35], and disassembly of the oligomeric complexes into individual components. The new membrane configuration and charge [17] could well favour the shuttling of one or more components to MEGF10, thereby increasing its pro engulfment function. Cytoskeletal motors or scaffolds such as dynamin [4] able to propel MEGF10 along the forming phagosome could well fulfil this role.

## Materials and Methods

### Northern Blot

Total RNA from tissues and cell lines was extracted following standard procedure by LiCl precipitation or by RNeasy extraction kit (Quiagen Inc, Hilden, Germany). Ten µg of total RNA were loaded on denaturing agarose gel and blotted overnight onto nitrocellulose membrane (Schleicher & Schuell, Dassel, Germany). Blots were probed with a MEGF10 fragment (nt 3036-3287, Genbank #AB058676) labelled with the prime-a-gene labelling kit (Promega, Madison, WI, USA). For quantitative comparison, blots were simultaneously hybridized with an actin probe.

### Plasmid generation

pf01012 (MEGF10 cDNA, Genbank #AB058676) was obtained from the IMAGE consortium and cloned into the XhoI-AgeI sites of pECFP-N1, pEGFP-N1, pEYFP-N1 (Clontech, Palo Alto, CA, USA). MEGF10 APxA mutant (bearing N928A and Y931A mutations in the NPxY motif) was generated using the Quikchange XL mutagenesis kit (Stratagene, La Jolla, CA, USA). The P*_ced-1_ megf10::gfp* fusion construct was generated by cloning *megf10* cDNA and P*_ced-1_*, the promoter for *ced-1* into the *C. elegans* expression vector pPD95.75 [18].

pBI ABCA1 EYFP was described previously [36]. pBI ABCA1MM EYFP was generated by replacing the BsrGI-BsrGI fragment of pBI ABCA1 EYFP by the corresponding fragment containing the MM mutation [14].

Mouse ABCA7 cDNA was reconstituted from original lambda clones [26] by Overlap Extension-PCR (OE-PCR) amplification [37] and cloned into the NotI-SalI sites of pBI (Clontech, Palo Alto, CA, USA) or pBud CE4.1 vector (Invitrogen, Carlsbad, CA, USA). The EYFP chimera was similarly generated by OE-PCR and subsequently cloned by replacing the fragment of interest in pBI ABCA7. ABCA7MM EYFP was generated by replacing the Acc65I-Acc65I fragment of ABCA7 by the same fragment bearing the K→M mutation in each walker A motif (K844 and K1847) introduced by OE-PCR.

CD36 cDNA was kindly provided by Chris Gregory. CD36 EYFP fusion was generated by OE-PCR and cloned into the pBud CE4.1. To generate the CD36 ECFP fusion we adapted the sequence swapping protocol proposed by the Quikchange XL mutagenesis kit [38]. All constructs were validated by sequencing (MWG Biotech, Ebersberg, Germany).

### Cell transfection

Transient transfections were performed on 60% confluent monolayers of HeLa Tet off cells, cultured in DMEM medium, containing 10% FCS (GIBCO BRL, Gaithersburg, MD, USA) with a total of 5 µg of plasmid DNA mixed to EXGEN 500 (Euromedex, Mundolsheim, France) accordingly to manufacturer's instruction and as described previously [14]. The mix was left in contact with cell monolayers for 18 h and cells seeded according to experimental needs 24 h after transfection. Transfection efficiency, monitored when appropriate by flow cytofluorometry on a FACScalibur (Becton Dickinson, Mountain view, CA, USA) 24 h after transfection, was consistently at around 40%. In the case of ECFP chimeras, transfection efficiency was monitored visually by confocal microscopy examination. In the case of multiple simultaneous transfections, the total amount of DNA was kept invariant (5 µg) and the ratio between the DNA species varied as mentioned. Titration of the protein products was confirmed by fractionation on SDS-PAGE, blotting and hybridization with anti-ABCA1 (mAb 891.3 – [25]) or anti-GFP antibodies (clone 7.1/13.1, Roche Diagnostics, Mannheim, Germany). Cells were further analyzed for FRET or biochemical assays 60 h after transfection.

### Surface biotinylation and protein analysis

Surface biotinylation was carried out as described [36] on 3–5×10^5^ cells with 1 mg/ml NHS-LC-biotin (Pierce, Rockford, IL, USA) in ice cold PBS for 30 min, lysed in 100 mM TrisHCl pH 8, 100 mM NaCl, 10 mM EDTA, 1% Triton X-100 for 30 min at 4°C. Normalized amounts of cell lysates were immunoprecipitated with the anti-GFP antibody, according to standard protocols, loaded on SDS-PAGE and blotted onto nitrocellulose membrane. The biotinylated proteins were probed by streptavidin-HRP followed by revelation with ECL detection reagent (Amersham Pharmacia Biotech, Uppsala, Sweden).

### *In vitro* phagocytosis assay

*In vitro* engulfment assays were performed as described previously [14] 48 h after transfection. Cells seeded on microscopic slides were incubated in DMEM 5% FCS for 2 hours in the presence of excess of irradiated (600 rad) thymocytes, previously labelled for 20 min at RT with 7-AAD (20 µg/ml in PBS – Sigma Aldrich, St. Louis, Missouri, USA). After extensive washing to remove unengulfed thymocytes, cells were fixed with 4% paraformaldehyde in PBS pH 7.4. Slides were then mounted in Mowiol for observation by confocal microscopy (LSM 510 on Axiovert 200 inverted microscope – Zeiss, Oberkochen, Germany). The number of ingested thymocytes per cell was visually scored on a sample ≥100 transfected cells. Results were expressed either by plotting the fraction of cells having ingested a given number of thymocytes or as phagocytic index (average number of apoptotic thymocytes per transfected cell). Statistical analyses (Paired student t-tests) were performed using GraphPad Prism software (GraphPad Software, Inc., San Diego, USA).

### GST pull down experiments

GST pull down were performed as described [11]. Briefly, transfected HeLa cells were cultured for 48 hours before lysis in 10 mM TrisHCl, pH 7.4, 150 mM NaCl, 1 mM EDTA, 1% Triton X-100, 0.5% NP-40 plus protease inhibitors. Ten µg of bacterially produced GST or GST-GULP fusion proteins were immobilized on beads and incubated with cell lysates for 4 hours at 4°C. After extensive washes, the bound proteins were fractionated on SDS-PAGE and immunoblotted using the anti-GFP antibody. GST fusion proteins were revealed by Ponceau S staining of the membrane.

### Imaging FRET measurements

FRET was measured by the method of acceptor photobleaching [28], [29], [39] by a Zeiss LSM 510 module mounted on an Axiovert 200 inverted microscope (Zeiss, Oberkochen, Germany). The microscope was equipped with a 25 mW Argon/2 laser beam and a polychromatic multichannel detector (META detector) to spectrally resolve emission spectra. ECFP and EYFP were illuminated respectively with the 458 (60% intensity of the acoustico-optical tunable filter – AOTF) and 514 nm (6% intensity of the AOTF) laser lines. To maximize selectivity, 4 dimension stacks were acquired in spectral mode with wavelength series at 10-nm intervals recorded for every time series. EYFP and ECFP images were subsequently reconstituted by linear unmixing of the wavelength series. Regions of interest (ROI) corresponding to membrane colocalization of the two fluorochromes were selected visually on images acquired on double transfected cells. ROI were bleached at 514 nm (100% intensity of AOTF) by 400 reiterations as previously calibrated [40]. Bleaching time varied from 150 to 200 sec. A set of 5 images was acquired before and after bleaching in spectral mode and reconstituted by linear unmixing to visually check both bleaching efficiency and stability of the sample. FRET efficiency was calculated according to the formula E_f_ = I_post_−I_pre_×100/I_post_ on numerical values tabulated by the LSM software. Background levels, measured as pixel values outside cells or in the cytoplasmic region, were computed in each experimental set and never exceeded 20% of signal.

### Native PAGE fractionation and analysis

Monolayers of HeLa cells (60 hours after transfection with the indicated plasmid), were rinsed in PBS before solubilisation in 1% α-DM, 150 mM NaCl and 50 mM TrisHCl, pH 7.4 for 30 minutes at 4°C. Homogenates were then spun at 4°C at 100,000×g for 20 min to eliminate aggregates and protein concentration in the cleared post-nuclear supernatant determined. Between 5 and 20 µg of proteins were loaded on a Deriphat/PAGE system adapted from Peter and Thornber [30] and analysed as previously described [25]. After electrophoresis at 4°C at 50 Volt for 12 hours, proteins were electrically transferred onto nitrocellulose membrane. Membranes were subsequently probed with the anti ABCA1-891.3 mAb or anti-GFP mAb followed by the appropriate secondary antibody and revealed by ECL detection reagent. The migration of native protein standards (Amersham Biosciences, Saclay, France) was analyzed by Ponceau S staining of the membrane.

### *C. elegans* ectopic expression of MEGF10

The MEGF10 EGFP fusion construct was introduced as extra-chromosomal array into *ced-1* complete loss-of-function mutants *ced-1(e1735)* and into the wild-type background. Transgenic lines were obtained using standard microinjection technique and transgenic animals identified as GFP^+^ by fluorescence microscopy. Engulfment defects were measured by counting the number of persistent cell corpses which appear as highly refractile, button-like objects in the head region of newly hatched L1 larvae (hatched within 2 hours) using the Nomarski Differential Interference Contrast Microscopy. L1 Larvae were anesthetized with 30 mM NaN_3_ and mounted on agar pads before counting.
